# Evaluation of the use of a novel bioabsorbable polymer drug-eluting microsphere for transarterial embolization of hepatocellular neoplasia in dogs

**DOI:** 10.1371/journal.pone.0269941

**Published:** 2022-08-08

**Authors:** William T. N. Culp, Eric G. Johnson, Michelle A. Giuffrida, Robert B. Rebhun, James K. Cawthra, Heidi A. Schwanz, Jenna H. Burton, Michael S. Kent

**Affiliations:** 1 Department of Veterinary Surgical and Radiological Sciences, University of California-Davis, School of Veterinary Medicine, Davis, California, United States of America; 2 Boston Scientific Corporation, Marlborough, Massachusetts, United States of America; Colorado State University, UNITED STATES

## Abstract

In dogs with non-resectable hepatic neoplasia, treatment options are limited. The objectives of this study were to describe the use of a novel drug-eluting embolic microsphere containing paclitaxel for use during transarterial chemoembolization (TACE), to compare results of liver-specific owner questionnaires and tumor volume pre- and post-TACE, and to measure systemic paclitaxel concentration post-TACE. Client-owned dogs with non-resectable hepatic neoplasia were prospectively enrolled. All owners completed questionnaires validated for the assessment of subjective outcomes in dogs with cancer before the TACE procedure and approximately 4 weeks after the TACE procedure. A CT scan was performed before TACE and 1 month after TACE; results were compared. Blood samples were obtained at specified time points post-TACE to determine systemic paclitaxel concentrations. Seven dogs (median weight: 8.9 kg; range, 4.3–31 kg) were enrolled. TACE was successfully performed in all dogs, and no intra-procedural complications were encountered. Questionnaire scores improved significantly post-TACE. Among the 6 dogs for which full data were available, median pre-TACE tumor volume was 390 cc (range 152–1,484; interquartile range 231–1,139) and median post-TACE tumor volume was 203 cc (range 98–889; interquartile range 151–369), which was significantly (P = .028) lower. All 6 dogs had a reduction in volume at the post-TACE measurement. Mean percent change in tumor volume was -45.6% (95%CI -58.6 to -32.6%). The mean plasma paclitaxel concentration in canine blood peaked at 4 days post-TACE procedure and was 25.7 ng/mL (range = 3.09–110 ng/mL) Median survival time was 629 days (95%CI 18 to upper limit not reached). The use of a novel paclitaxel-eluting microsphere in this cohort of dogs successfully decreased tumor volume significantly after TACE and improved clinical signs. Future investigation into the use of TACE and other similar therapies is warranted due to the promising outcomes noted in this cohort.

## 1. Introduction

Canine hepatic neoplasia can result in significant morbidity. Hepatocellular carcinoma (HCC) is the most common form of primary hepatic neoplasia and occurs in 3 major morphologic forms: diffuse, nodular and massive [1]. The massive form accounts for greater than 60% of HCC in dogs, and surgical resection is the treatment of choice, when possible [1, 2]. Other forms of HCC (nodular and diffuse) are generally not amendable to surgery, and massive forms of HCC that grow unchecked for a period of time, and surround or invade adjacent anatomical structures may be associated with high levels of surgical morbidity.

HCC is one of the most commonly diagnosed solid cancers in humans and is a common cause of death from malignancy [3]. Similar to dogs, surgical resection is the treatment of choice in humans; however, non-resectability is common [3]. For this reason, alternative therapies have been explored both experimentally and clinically and have shown some promising results [3–5].

One such alternative is the transarterial administration of embolic agents solely (transarterial embolization or TAE) or combined with chemotherapy (transarterial chemoembolization or TACE). These procedures involve the use of interventional radiologic techniques to deliver embolic agents directly to tumors to diminish or eliminate tumor blood supply [4–6]. Tumor embolization is generally performed via catheter-delivery of embolic agents. Several embolic agents have been utilized to perform embolization, and these agents are available in temporary and permanent forms; generally, the temporary embolic agents (eg. absorbable gelatin powder) are utilized for trauma or in situations when a permanent occlusion would cause unacceptable end-organ devascularization. Permanent agents commonly chosen for tumor embolization include particles such as polyvinyl alcohol and acrylic spheres. A novel drug-eluting microsphere (Oncosphere Embolic Microsphere™, Boston Scientific, Marlborough, MA) that is a mixture of poly (lactic-co-glycolic acid), or PLGA, and paclitaxel has been developed. The drug-eluting microsphere regulates the release of paclitaxel to the surrounding tissue resulting in extended drug release.

As the treatment options for dogs with hepatocellular neoplasia that is determined to be surgically challenging are limited, it is imperative that alternative therapies be evaluated. Therefore, the aims of this investigative group are three-fold: 1) to describe the procedure and technical outcome of transarterial chemoembolization (TACE) with a novel chemoembolic agent (paclitaxel-eluting microspheres), 2) to evaluate the effect that TACE has on clinical signs (as determined by an owner questionnaire) and tumor volume (as measured by computed tomographic imaging), and 3) to evaluate the systemic concentration of paclitaxel at varying time points and record toxicity associated with chemotherapy administration in a group of dogs with naturally-occurring hepatocellular neoplasia. The results of this study demonstrated that TACE was technically feasible in dogs with hepatocellular neoplasia utilizing a novel microsphere, dogs undergoing TACE demonstrated an improvement in clinical signs and a decrease in tumor volume, and paclitaxel delivered in this manner was well tolerated and able to be measured.

## 2. Materials and methods

### 2.1 Animals and procedure

Client-owned dogs with naturally-occurring hepatocellular neoplasia that was determined to be non-resectable (via previous surgical exploration) or in a location with great surgical risk were prospectively enrolled in the clinical trial from May 2017 until March 2019. The study protocol was explained to the owner, and informed consent was obtained prior to initiation of treatment; as part of this consent, owners consented to enrolling their dog in this study, allowing their dog to undergo the diagnostics and treatments described below and providing information in previously validated questionnaires. A cytologic or histopathologic diagnosis of hepatocellular neoplasia (either carcinoma or adenoma) was required. This study was approved by the Institutional Animal Care and Use Committee (Protocol #: 19518). Animals were excluded if any of the following criteria were identified: determination of potential surgical resectability after CT scan, receiving chemotherapy within 2 weeks prior to TACE or receiving corticosteroids or NSAIDs within 1 week prior to TACE. All dogs undergoing TACE were monitored closely for any pain associated with the procedure, and appropriate analgesia was administered as necessary.

Procedural-related adverse events were recorded as reported by Follette et al. [7] Procedural time and time to discharge were recorded. “Technical success” of the procedure was defined as the following: 1. the ability to access the tumoral blood supply, specifically at the level of the hepatic arterial branch (arterial branch off the hepatic artery after branching of the gastroduodenal artery, and -at least a 3^rd^ order arterial branch from the aorta) providing arterial blood supply only to the tumor tissue, and 2. the administration of the chemoembolic agent to the tumoral blood supply. Following TACE, all dogs were administered amoxicillin with clavulanic acid (14–20 mg/kg) orally two times daily for 30 days. Additionally, gastrointestinal protectants such as proton pump inhibitors, serotonin receptor antagonists, and selective neurokinin 1 receptor antagonists were administered as needed.

The procedure was performed as follows: All dogs were placed under general anesthesia utilizing an individualized protocol as determined by the clinical anesthesiology service and then transported to the fluoroscopy (GE OEC 9900 Elite, GE Healthcare, Chicago, IL) suite. Each dog was placed in dorsal recumbency, and the groin region clipped, prepared with aseptic technique and draped. All TACE procedures were performed by a single author (WTNC). A 1–2 cm incision was made over the femoral artery, and the subcutaneous tissues were bluntly and sharply dissected until the artery was visualized. Two 3–0 polydioxanone sutures (PDS^®^, Ethicon US, LLC, Bridgewater, NJ) were placed around the femoral artery. An 18-gauge over-the-needle catheter (Becton, Dickinson and Company, Franklin Lakes, NJ) was introduced into the femoral artery and the needle was removed. An 0.035” hydrophilic guidewire (Weasel Wire^®^, Infiniti Medical, Redwood City, CA) was introduced into the over-the-needle catheter and subsequently into the femoral artery. The over-the-needle catheter was removed over the guidewire. A vascular access sheath and dilator (Introducer Sheath and Dilator, Infiniti Medical, Redwood City, CA) were introduced into the femoral artery over the guidewire. The dilator was then removed over the guidewire. The fluoroscopy unit was then positioned over the abdomen of the patient. A 4 French hook catheter (Cobra Catheter, Infiniti Medical, Redwood City, CA) was placed over the guidewire, and the guidewire/hook catheter combination was passed from the femoral artery through the external iliac artery to the abdominal aorta, and the celiac artery was subsequently selected. Once in the celiac artery, the main hepatic artery was selected. The guidewire was then removed from the vascular access sheath. A 50% saline/50% contrast medium (Isovue-370, Bracco Diagnostics Inc., Princeton, NJ) mixture was drawn into a syringe and attached to the hook catheter and injected under fluoroscopic guidance to perform an angiogram and identify the tumoral blood supply. During this angiogram, injected contrast medium was monitored closely to be sure that hepatopulmonary shunting was not occurring; lack of shunting was confirmed in all cases. Superselection of the hepatic arterial blood supply to the tumor was then performed with a combination of a microwire (Fathom™ 14 Microwire, Boston Scientific, Marlborough, MA) and microcatheter (Renegade™ 14 Microcatheter, Boston Scientific, Marlborough, MA).

Drug-eluting bead TACE was performed (without ethiodized oil). The chemoembolic agent used was a bioabsorable polymer microsphere (75–100 μm diameter) loaded with paclitaxel (10% paclitaxel) (Oncosphere Embolic Microsphere™, Boston Scientific, Marlborough, MA). The syringe containing the paclitaxel-loaded microspheres was prepared. The microspheres were hydrated in saline; all but 1 ml of saline was then removed. Then, 8 ml of contrast medium was transferred to the syringe with the microspheres and transferred back and forth several times until appropriate suspension was noted. The created slurry of microspheres was transferred into 1 ml syringes and administered to the tumoral blood supply, being injected under fluoroscopic guidance. The slurry was injected in short bursts, and the flow of the slurry through the vessel being embolized was monitored closely to prevent retrograde flow. When cessation of the flow was documented fluoroscopically, the injection was discontinued. Selective and non-selective arteriography were then performed to assess the efficacy of the procedure. Additionally, arteriography was performed to ensure patency of the main hepatic artery and celiac artery. Upon completion of the arteriograms, the hook catheter was removed over the guidewire. The vascular access sheath was then removed from the femoral artery and the previously placed ligatures were tightened around the vessel. The subcutaneous tissue and skin were closed routinely.

### 2.2 Questionnaires and clinical signs

All owners completed questionnaires validated for the assessment of subjective outcomes in dogs with cancer before the TACE procedure and approximately 4 weeks after the TACE procedure. The questionnaires consisted of: the Canine Symptom Assessment Scale with a novel 4-item liver subscale (CSAS-Liver) and the Canine Owner-Reported Quality of Life Scale (CORQ). The CSAS is multi-dimensional symptom assessment tool that measures frequency, severity, dog distress and owner distress associated with 12 general and 4 liver-specific signs an owner might have observed during the preceding 10-day period; higher CSAS scores indicate greater symptom burden. The CORQ is a 17-item questionnaire that measures general quality of life as observed by the owner during the preceding week; higher scores indicate better quality of life.

### 2.3 Clinical laboratory assessment

A pre-TACE complete blood count (CBC) and biochemical panel were performed in all dogs. Additionally, a biochemical panel was obtained 1 day after TACE and a CBC was obtained approximately 1 week after TACE. A CBC and biochemical panel were obtained approximately 4 weeks after TACE.

### 2.4 Imaging: Computed tomography scans

A dual phase (arterial and venous) contrast-enhanced CT scan (GE Lightspeed 16, GE Medical Systems, Milwaukee, WI) of the liver was performed prior to TACE and approximately 4 weeks after TACE. The CT scan was performed using a clinical protocol of overlapping (0.5:1) 2.0mm thick helical images prior to and after a systemic dose of iodinated contrast media. The images were reconstructed in soft tissue and bone algorithms. Tumor volumes were calculated by a single author who was blinded to study data using a commercially available imaging analysis software package (Osirix v4.1.2, Pixmeo, Bernex, Switzerland). Briefly, contrast-enhanced CT images were imported into the software package and a freeform tool was used to draw a line around the tumor in a single image to create a region of interest (ROI). When needed, a repulsor tool was used to specifically align the ROI to only include the tumor tissue. This same procedure was performed on sequential images until the entire tumor was outlined. All ROIs were then selected, and a software tool for computing the tumor volume was utilized. The pre-TACE tumor volumes were compared to the 4-week post-TACE tumor volumes.

### 2.5 Blood sampling and paclitaxel concentrations

Blood samples were obtained pre-treatment and at the following time points to allow for determination of intravascular paclitaxel concentration: 15 minutes, 1 hour, 4 hours, 8 hours, 24 hours, 4 days, 7 days, and 14 days after administration. Whole blood was obtained and placed in an EDTA tube. Blood samples were then flash frozen and stored at -80°C. Blood analysis was performed with liquid chromatography–mass spectrometry (LC-MS) to quantify the concentration of paclitaxel in canine blood.

### 2.6 Adverse events

An adverse event (AE) post-TACE was categorized as either “chemotherapy-related” or “procedure-related”. Adverse events considered to be chemotherapy-related were reported and graded according to VCOG guidelines [8]. Adverse events considered to be procedure-related were classified according to the Society of Interventional Radiology Adverse Event Classification scale, which has been specifically designed for human patients undergoing Interventional Radiology procedures such as hepatic chemoembolization [9]. Signs consistent with postembolization syndrome, or PES (ie. fever, leukocytosis, abdominal pain post-TACE, lethargy and/or nausea/vomiting) and increases in hepatocellular leakage enzymes were not considered AEs, as these are expected post-TACE [9]; however, those variables were recorded.

### 2.7 Statistical analysis

#### 2.7.1. Power analysis

The endpoint used for power analysis was the proportional difference between pre-procedure and post-procedure tumor volume (cc) as obtained with CT scans, according to the formula: (post-TACE—pre-TACE)/pre-TACE. A sample size of 10 dogs was estimated to have 80% power to detect a 50% change in post-TACE volume as significantly different from zero change, assuming a SD of 50% and alpha = .05.

#### 2.7.2. Statistical plan

The primary numerical endpoints to be analyzed were the volume measurements obtained during the CT scans. Secondary outcomes included describing and comparing clinical signs, AEs and quality of life before and after TACE, as measured via questionnaires. The relationship between CORQ and CSAS-Liver scores before and after TACE was estimated using Spearman rank correlation coefficients. CORQ scores (potential range 0–7), CSAS-Liver scores (potential range 0–64), and tumor volume measurements were compared before and after TACE using Wilcoxon signed rank tests. Mean score differences and percent change in tumor volume between time points were estimated with surrounding 95% confidence intervals (CI) using the Clopper-Pearson exact binomial method. Median survival time following TACE was estimated using the Kaplan-Meier method; dogs alive or lost to follow up were censored in the analysis. All tests were two-sided and P < .05 was statistically significant. Statistical analysis was performed using a commercially-available program (Stata version 14.2, College Station, TX).

## 3. Results

### 3.1 Animals and procedural outcome

Prospectively, 7 dogs were enrolled in this trial. Study enrollment was closed early due to poor enrollment. Multiple other dogs were screened for the trial, but after CT evaluation, these dogs were considered to have liver tumors that were surgically resectable. The median age and weight of enrolled dogs were 12 years (range, 9–13 years) and 8.9 kg (range, 4.3–31 kg). The gender distribution was 4 male castrated and 3 female spayed dogs, and the breeds included mixed breed (n = 4) and 1 each of dachshund, golden retriever, and Jack Russell terrier.

The diagnosis of the liver masses included hepatocellular carcinoma (n = 6) and adenoma (n = 1). Tumor location and characteristics were as follows: right lateral and medial liver lobes and caudate lobe (n = 2), quadrate lobe (2), right lateral liver lobe and caudate lobe (with compression of caudal vena cava) (1), caudate lobe but involving common bile duct and portal vein (1), entire mid-liver affected, but could not characterize further (1). Tumors were considered either non-resectable or presenting too great of a surgical risk after surgical explore in 4/7 dogs and after CT in 3/7 dogs.

No intra-procedural complications were encountered, and technical success was considered 100% as all tumoral blood supplies could be accessed and microspheres were successfully administered. Median procedural time was 165 minutes (range, 105–195 minutes). Median days of hospitalization post-TACE was 3 (range, 1–14 days), and all dogs were discharged.

### 3.2 Questionnaires and clinical signs

The most common pre-TACE owner-reported clinical signs were lack of energy (5/7, 71.4%), large or swollen abdomen (4/7, 57.1%), difficulty sleeping (4/7, 57.1%), panting (3/7, 42.9%), increased drinking (3/7, 42.9%), and yelping or crying out (3/7, 42.9%). Moaning, groaning or whining was reported in 2/7 (28.6%) dogs, and pain, cough, lack of appetite, sleepiness, vomiting, and increased frequency of urination were each reported in 1/7 (14.3%) dogs. Seizures, diarrhea, and pacing were not reported in any dog. Median preoperative CSAS-Liver score was 9.4 (range 0.0–19.2). Median pre-TACE CORQ score was 6.06 (range 4.82–6.88). There was a modest negative correlation (ρ = -0.43; P = .34) between preoperative CORQ and CSAS-Liver scores. One dog had no owner-reported clinical signs, one had 2 different clinical signs, one had 4 clinical signs, two had 5 clinical signs, and two had 7 clinical signs.

Four-week-post-TACE questionnaire results were available for 6 dogs. Pain, lack of energy, panting, and pacing were each reported in 2/6 (33.3%) dogs. Cough, lack of appetite, sleepiness, vomiting, diarrhea, and large or swollen abdomen were each reported in 1/6 (16.7%) dogs. Difficulty sleeping, moaning, groaning, or whining, yelping or crying out, seizures, increased drinking, and increased frequency of urination were not reported in any dog. Median 4-week post-TACE CSAS-Liver score was 0.0 (range 0.0–12.2). Median 4-week post-TACE CORQ score was 6.91 (range 5.82–7.00). There was a strong negative correlation (ρ = -0.90; P = .015) between 4-week post-TACE CSAS-Liver and CORQ scores. Four dogs had no symptoms, one dog had 5 different symptoms, and one dog had 9 symptoms.

All 6 dogs’ individual CSAS-Liver scores improved between pre- and post-TACE measurements, except the dog with a zero pre-TACE score also had a zero post-TACE score. Dogs’ postoperative CSAS-Liver scores were significantly (P = .035) lower than their preoperative scores, indicating lower postoperative clinical signs burden; the mean score difference was -5.8 (95%CI -11.0 to -0.2). Five of 6 dogs’ CORQ scores improved between preoperative and postoperative measurements, and one dog’s CORQ score worsened. The dog with the worsened score was the dog experiencing 9 different clinical signs. Dogs’ postoperative CORQ scores were not significantly different from their preoperative scores (p = .11); the mean score difference was +0.66 (95%CI -0.27 to 1.57).

### 3.3 Clinical laboratory assessment

Pre-TACE values are available for all 7 dogs (Tables 1 and 2); as 1 dog died 18 days after TACE, post-TACE values at the 4-week mark were not available. Pre-TACE, 3/7 dogs were anemic and approximately 4 weeks post-TACE, 1/6 was anemic. Pre- and approximately 4 weeks post-TACE, 5/7 and 5/6 dogs demonstrated thrombocytosis. One of 7 dogs was leukocytic pre-TACE with 3/7 demonstrating leukocytosis 1-week post-TACE; all dogs had normal WBC 4 weeks post-TACE. One of 7 dogs was neutrophilic pre-TACE with 3/7 demonstrating neutrophilia 1-week post-TACE and 1/7 demonstrating neutropenia; all dogs had normal neutrophil counts 4 weeks post-TACE.

**Table 1:** Complete blood count results before and after embolization in the dogs of this study.

Complete Blood Count				
Bloodwork Parameter	Time of Collection (in reference to embolization)	N	Median	Range
Hematocrit (%)	Pre	7	41.8	30.0–49.7
	4 weeks post	6	43.2	31.8–48.0
Platelets (/ul)	Pre	7	550,000	252,000–1,240,000
	4 weeks post	6	453,000	226,000–1,098,000
White blood count (/ul)	Pre	7	8,240	4,500–18,160
	1 week post	7	11,990	1,170–24,650
	4 weeks post	6	6,345	5,080–9,610
Neutrophils (/ul)	Pre	7	6,526	3,074–15,822
	1 week post	7	10,359	104–18,060
	4 weeks post	6	4,861	3,519–8,245

**Table 2:** Biochemical panel results before and after embolization in the dogs of this study.

Biochemical Panel				
Bloodwork Parameter	Time of Collection (in reference to embolization)	N	Median	Range
ALT (IU/L)	Pre	7	1,117	68–1,453
	1 day post	7	5,490	1,710–8,919
	4 weeks post	6	222	63–1,395
AST (IU/L)	Pre	7	48	35–230
	1 day post	7	4,541	272–8,748
	4 weeks post	6	58	24–486
Bilirubin (mg/dL)	Pre	7	0.2	0.2–0.3
	1 day post	7	0.2	0.2–0.5
	4 weeks post	6	0.2	0.2–0.2

At least one of the hepatocellular leakage enzymes was increased in every dog pre-TACE with elevations in ALT in 6/7 and AST in 3/7 dogs. The ALT increased 1-day post-TACE by a median of 1,496% (range, 391–2,414%), and the AST increased 1-day post-TACE by a median of 5,160% (range, 655–15,521%). ALT was decreased from pre-TACE values in 5/6 dogs and AST was decreased from pre-TACE in 3/6 dogs at approximately 4 weeks post-TACE. In the dogs with decreased ALT and AST post-TACE, the ALT decreased 4 weeks post-TACE (as compared to pre-TACE) by a median of 42% (range, 7–67%), and the AST decreased 4 weeks post-TACE (as compared to pre-TACE) by a median of 31% (range, 19–41%). Bilirubin, which was normal in all dogs pre-TACE, increased above normal (0.3 and 0.5 mg/dL, respectively; reference range: 0.0–0.2 mg/dL) in 2/7 dogs 1-day post-TACE and was normal in all dogs 4 weeks post-TACE.

### 3.4 Imaging

**Fig 1:**
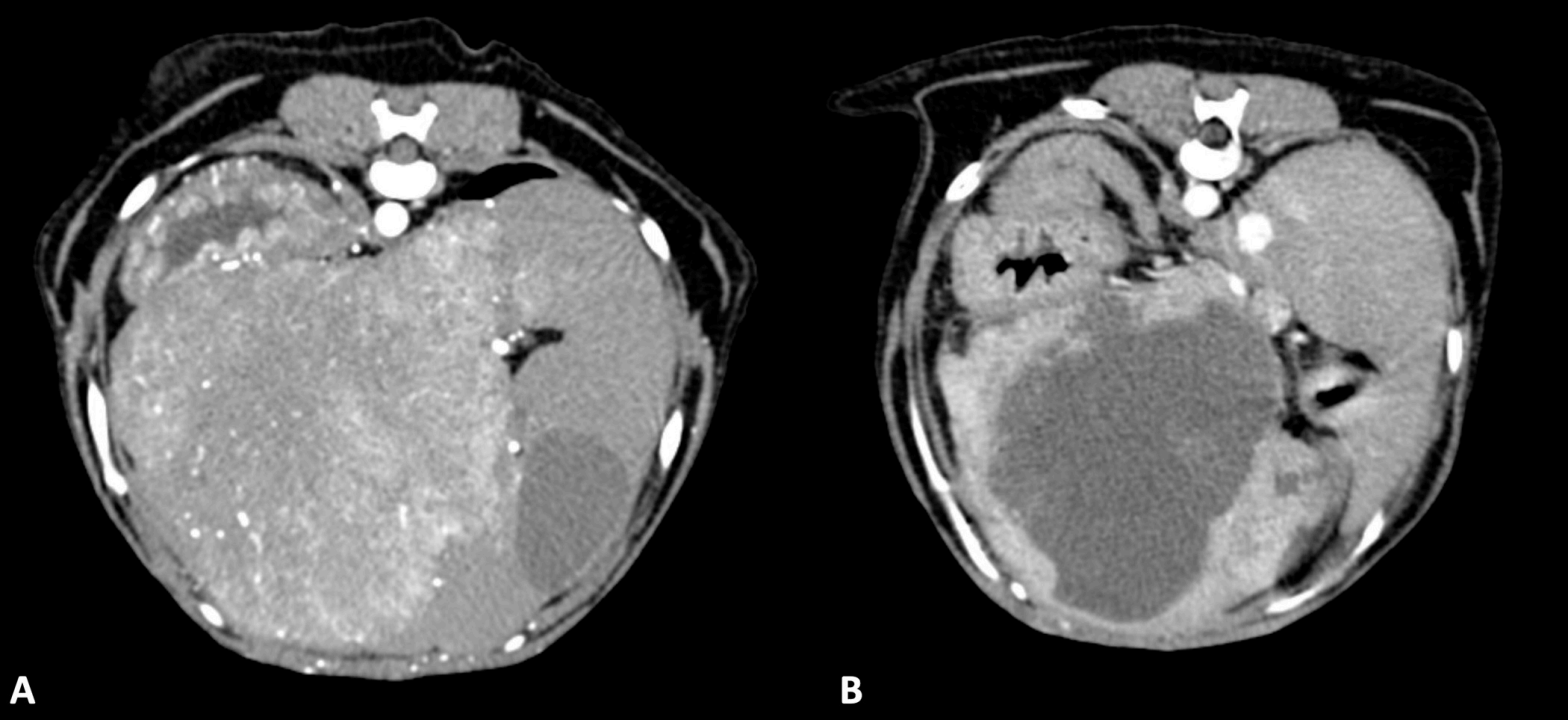
Transverse abdominal CT images from a 10-year-old FS mixed breed dog. (A) CT image of a hepatocellular carcinoma pre-embolization and (B) 4-weeks post-embolization.

All dogs underwent a CT scan within 1-day prior to TACE, and 6 of 7 dogs underwent a 4-week post-TACE CT scan (Fig 1). Among the 6 dogs for which full data were available, median pre-TACE tumor volume was 390 cc (range 152–1,484; interquartile range 231–1,139) and median post-TACE tumor volume was 203 cc (range 98–889; interquartile range 151–369), which was significantly (P = .028) lower. All 6 dogs had a reduction in volume at the post-TACE measurement. Mean percent change in tumor volume was -45.6% (95%CI -58.6 to -32.6%) at approximately 4 weeks after TACE.

### 3.5 Blood sampling and paclitaxel concentrations

The median dose of paclitaxel administered during TACE was 87 mg/m^2^ (range, 39–147 mg/m^2^). The mean plasma paclitaxel concentration peaked at 4 days post-TACE procedure and was 25.7 ng/mL (n = 7; range = 3.09–110 ng/mL). After 14 days, the mean plasma paclitaxel concentration had decreased to 2.39 ng/mL (n = 7; range = 0.289–5.54 ng/mL).

### 3.6 Adverse events

Following TACE, 2/7 dogs exhibited no chemotherapy- or procedure-related AEs. One dog experienced a grade 4 neutropenia (104/uL) 1-week post-TACE. The dog was continued on the amoxicillin and clavulanic acid that had been previously prescribed and monitored for any other complications. At 2 weeks post-TACE (1-week later), the neutrophil count in that dog was 17,716/uL.

**Fig 2:**
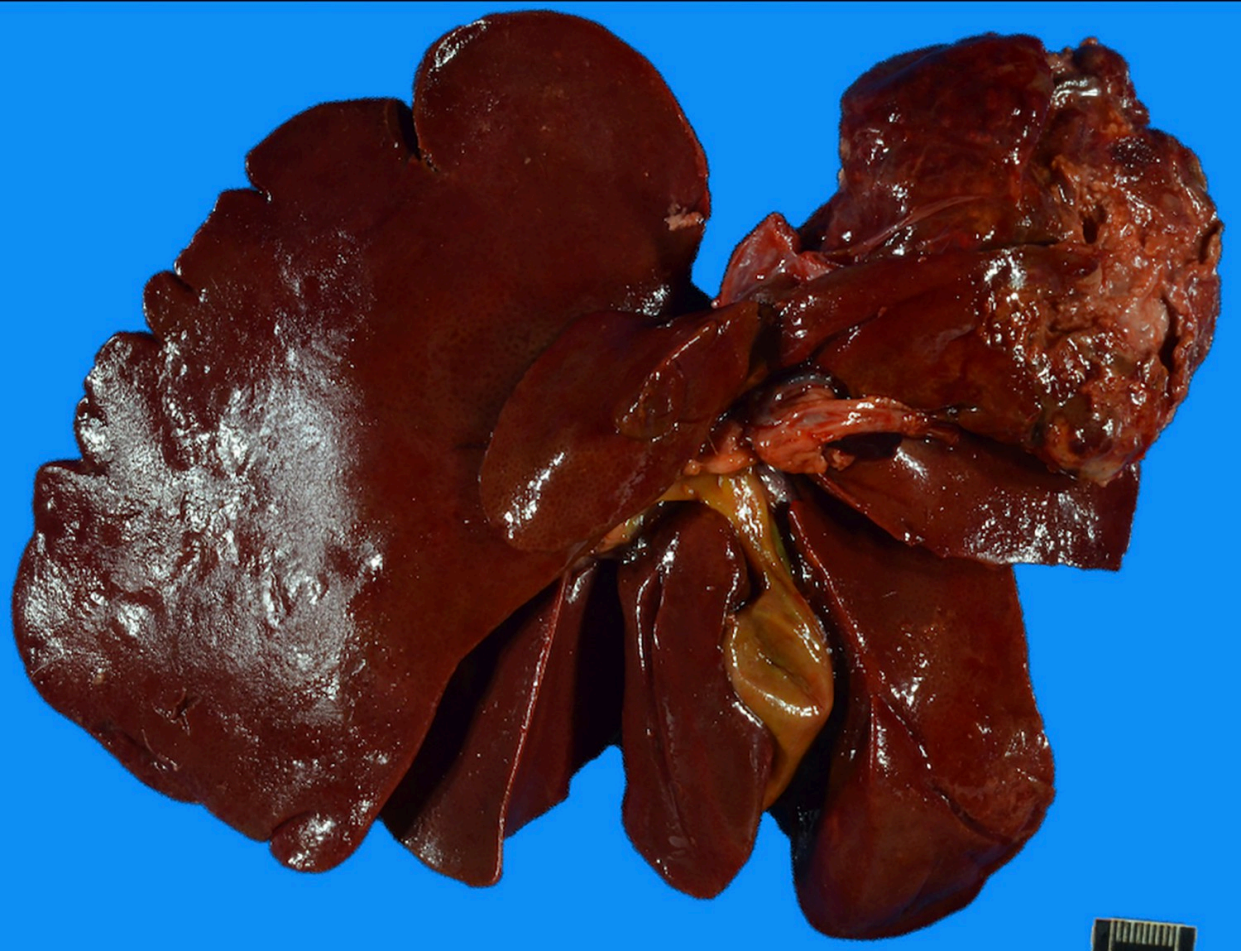
Necropsy image of the liver of a 12-year-old Jack Russel terrier.

For procedure-related AEs, 1 dog demonstrated a level 2 AE, and 1 dog demonstrated a level 5 AE. Abdominal ultrasonography of the dog with the level 2 AE demonstrated that the pancreas was hypoechoic and enlarged with surrounding hyperechoic peripancreatic fat indicating pancreatitis and regional peritonitis. The dog was hospitalized for fluid administration and monitoring for 7 days; at 10 days post-TACE, the dog was free from clinical signs. In the dog with a level 5 AE, clinical signs resolved 7 days after TACE, and the dog was discharged. However, at 12 days post-TACE, the dog was evaluated after the owner noted the abdomen to be distended. During abdominal ultrasonography, the pancreas was noted to be hypoechoic and enlarged with surrounding hyperechoic peripancreatic fat indicating pancreatitis and regional peritonitis. Additionally, space-occupying material was identified in the aorta and left external iliac arteries indicating vascular thrombosis. The hepatic mass was hypoechoic, devoid of power Doppler signal and surrounded by echogenic fat suggesting necrosis and secondary regional inflammation. The dog was hospitalized for 1 day and discharged as he improved clinically. However, he was reevaluated 6 days later (18 days post-TACE) for worsening signs of abdominal distension and ataxia in the hind limbs. Hospitalization and anti-thrombotic treatment were recommended; however, the owner declined and instead elected humane euthanasia. On necropsy, extensive necrosis of the tumor and capsular rupture were noted (Fig 2) as was severe pancreatitis and aortic and left iliac arterial thromboembolism. The hepatic rupture was closely associated with the pancreas.

This necropsy image is of the liver of a dog with a hepatocellular adenoma who underwent transarterial embolization and was euthanized 18 days after the procedure. The liver mass is very necrotic and has ruptured contributing to the constellation of clinical signs noted in this dog.

Suspected PES signs were demonstrated in 5/7 dogs. Lethargy and hyporexia were noted post-embolization in 4/7 dogs, and 1 dog appeared nauseous; additionally, soft stool and fever were noted in 1 dog each. All signs were noted within the first 1–1.5 days post-TACE. Dogs were no longer exhibiting PES signs at a median time of 7 days (range, 0–14 days) after TACE, although some clinical signs (as noted on the CSAS-Liver score) were noted at approximately 4-weeks post-TACE.

### 3.7 Outcome

At the time of data analysis, 4 dogs had died at 18, 248, 349, and 629 days post-TACE, and 3 dogs were alive at 514, 745, and 878 days post-TACE. Median survival time was 629 days (95%CI 18 to upper limit not reached).

## 4. Discussion

Dogs undergoing treatment of hepatocellular neoplasia via a novel drug-eluting microsphere experienced a significant improvement in clinical signs based on assessment via questionnaire and significant decrease in tumor volume. The treatment was well-tolerated with minimal adverse events, high hospital discharge rate and prolonged survival times. Additionally, the technical success was high with low procedure-related complications, although one dog experienced a Grade 5 toxicity.

The use of TAE and TACE is particularly attractive for the treatment of hepatocellular neoplasia due to the dual blood supply of the liver. More specifically, normal liver parenchyma is supplied primarily by the portal vein whereas liver tumors receive blood supply almost exclusively from the hepatic artery [10, 11]. Therefore, despite embolization of the hepatic arterial blood supply, sufficient blood supply to the normal liver remains intact via the portal vein.

The use of hepatic embolization/chemoembolization in companion animals has been described retrospectively in a few reports; these studies have included a small number of dogs and both bland embolization and chemoembolization were described [12–14]. In one study where tumor volumes were calculated, all dogs experienced a decrease in postoperative tumor volume [14]. More recently, 2 prospective studies have described the use of drug-eluting bead transarterial chemoembolization (DEB-TACE) in dogs, and the outcomes associated with beads containing cisplatin [15] and doxorubicin [16], respectively, were reported. In the doxorubicin DEB-TACE study, stable disease or partial response of the tumor was reported in 85% of dogs [16].

The decision-making regarding the addition of chemotherapy to embolization and the means by which chemotherapy is administered (ie. conventionally mixed with beads or via a drug-eluting vehicle) remains controversial in human medicine; there is a paucity of information in veterinary patients and the best means of performing hepatic embolization has not yet been elucidated. The use of DEB-TACE in the treatment of liver neoplasia is commonplace in humans. DEB-TACE has been shown to be at least as equally effective as conventional TACE, but has been promoted as an alternative due to the potential for being a safer means of chemotherapy delivery [17]. When delivered via DEB-TACE, chemotherapy is slowly released which allows for greater local concentration of drug and less systemic exposure of drug.

The most common drug utilized in humans for DEB-TACE is doxorubicin. While several studies document the use of paclitaxel in the treatment of different canine tumors, this drug is still rarely utilized clinically [18]. Paclitaxel was highly tolerated in this group of dogs with minimal chemotherapy-related adverse events, although the median delivered dose is lower than what is generally administered intravenously (165 mg/m^2^) to dogs. Additionally, it is important to note that the drug administered in this study was paclitaxel without the addition of Cremophor^®^, which has been shown to cause hypersensitivity reactions in dogs regularly [18]. The mean peak concentration of paclitaxel systemically was noted 4 days post-TACE, with paclitaxel nearly gone at the 2-week post-TACE testing time. The delivery of chemotherapy via DEB theoretically decreases the exposure of the non-target organs to the effects of chemotherapy (hopefully decreasing systemic signs), and the systemic concentrations of paclitaxel reported in this study provide a basis for comparison for patients undergoing DEB-TACE in the future.

While it can be challenging to separate adverse events experienced secondary to chemotherapy from PES, the PES clinical signs encountered in this cohort of dogs was similar to what has historically been encountered in the authors’ clinic associated with bland embolization. The signs of PES in this cohort were common with 5 of 7 dogs demonstrating either lethargy, changes in appetite or gastrointestinal signs. Very little is understood about how PES manifests in companion animals, but based on the results of this small cohort, signs appear to be similar to what is expected in human patients undergoing liver TACE.

One of the 7 dogs treated in this study died in the short-term at 18 days post-TACE. The signs demonstrated by this dog are likely secondary to the significant response experienced by the tumor and the subsequent inflammatory cascade induced by hepatic necrosis, pancreatitis or both. On necropsy, pancreatitis was likely influenced by the close proximity to the affected (and subsequently treated) region of liver. Pancreatitis is a major concern whenever performing TACE in dogs; part of the blood supply to the pancreas branches from the gastroduodenal artery which is a branch directly off the main hepatic artery, and this close association could lead to non-target embolization. Additionally, when large right-sided liver tumors are encountered, it is not unusual for the pancreas to be adjacent or adhered to these tumors, and inflammation induced in the liver from treatment could similarly occur in the pancreas.

Clinical laboratory evaluation associated with treatment in this cohort of dogs produced some interesting results. Hepatocellular leakage enzymes (ALT and AST) were increased in most dogs pre-TACE; however, a dramatic increase in these enzymes was noted the day after TACE. This is an expected finding due to the nature of the treatment and the subsequent trauma caused to the hepatic tissue after TACE. Interestingly, the values at approximately 4-weeks post-TACE were improved from the pre-TACE values in most dogs.

While the outcomes reported here are only for a small number of dogs, the median survival times encountered in this cohort are encouraging. Four of the 6 dogs that survived the short term, survived greater than a year and 2 survived greater than 2 years (with an additional dog still alive at study completion at 514 days post-TACE). Comparatively, dogs with massive hepatocellular carcinoma that did not undergo treatment in a separate study had a median survival time of 270 days [2].

There are several limitations of this study that should be recognized. First, case numbers in this study were low; however, a benefit of the study design for this trial was that each treated dog could act as its own control due to evaluation of clinical signs and tumor volume pre- and post-TACE. Additionally, this procedure is technically challenging and requires fluoroscopy and exposure to chemotherapy, which increases the risk to both the dog and the clinicians involved in treatment. Lastly, this study did not perform comparisons with other available treatments. Other treatments have questionable efficacy when extensive liver neoplasia is encountered; however, further investigation is needed into how the outcomes associated with these different available options may compare.

The use of a novel paclitaxel-eluting microsphere in this cohort of dogs successfully decreased tumor volume significantly approximately 4-weeks post-TACE and improved clinical signs. The technique described here was well-tolerated with minimal AEs and was able to be performed in a minimally invasive fashion. Future investigation into the use of DEB-TACE and other similar therapies is warranted due to the promising outcomes noted in this cohort, and the current lack of other effective treatment options in dogs with extensive hepatocellular neoplasia.

## Supporting information

S1 Data (XLSX)
